# Regulatory mechanism of circular RNA involvement in osteoarthritis

**DOI:** 10.3389/fsurg.2022.1049513

**Published:** 2023-01-06

**Authors:** Yuke Zhang, Liting Liu, Kai Liu, Meiying Wang, Xiulan Su, Jianzhong Wang

**Affiliations:** ^1^ Inner Mongolia Medical University, Hohhot, China; ^2^ Department of Clinical Laboratory, The Affiliated Hospital of Inner Mongolia Medical University, Hohhot, China; ^3^ Clinical Medicine Research Center, The Affiliated Hospital of Inner Mongolia Medical University, Hohhot, China; ^4^ Department of Orthopedics and Traumatology, The Second Affiliated Hospital of Inner Mongolia Medical University, Hohhot, China

**Keywords:** osteoarthritis, circular RNA, cartilage degeneration, competitive endogenous RNA, RNA binding protein, exosomes, N6-methyladenosine

## Abstract

Osteoarthritis (OA) causes joint pain, stiffness, and dysfunction in middle-aged and older adults; however, its pathogenesis remains unclear. Circular RNAs (circRNAs) are differentially expressed in patients with OA and participate in a multigene, multitarget regulatory network. CircRNAs are involved in the development of OA through inflammatory responses, including proliferation, apoptosis, autophagy, differentiation, oxidative stress, and mechanical stress. Most circRNAs are used as intracellular miRNA sponges in chondrocytes, endplate chondrocytes, mesenchymal stem cells, synoviocytes, and macrophages to promote the progression of OA. However, a small portion of circRNAs participates in the pathogenesis of OA by intracellular mechanisms, such as protein binding, methylation, or intercellular exosome pathways. In this sense, circRNAs might serve as potential novel biomarkers and therapeutic targets for OA.

## Introduction

Osteoarthritis (OA) is one of the most common joint diseases that result in chronic pain and limited movement in the elderly (1). OA is characterized by the degradation of articular cartilage, subchondral bone sclerosis, osteophyte formation, and chronic synovitis, and is clinically characterized by slow progressive pain, stiffness, and dysfunction in the joints, seriously affecting the quality of life of patients (2, 3). Non-steroidal anti-inflammatory drugs are just an effective means of symptom control, while joint arthroplasty is the first line of treatment for patients with advanced disease. However, the early diagnostic biomarkers remain unexplored (4). Therefore, there is a need to explore the mechanisms underlying the development of OA, identify new biomarkers, and provide a theoretical basis for clinical and transformational research. Circular RNA (circRNAs) are highly conserved and tissue-specific non-coding RNA widely distributed in eukaryotic cells (5). They play important roles in regulating important cellular functions, by acting as miRNA sponges, thus regulating transcription, self-translation, and interacting with proteins (6). With the rapid development of high-throughput sequencing technology, many circRNAs associated with OA have been identified. CircRNAs are expressed differently in OA-related tissues and cells, further suggesting that circRNAs could be potential markers and targets for OA (7). Chondrocytes are cellular components of joint cartilage. Their involvement in inflammation-induced chondrocyte degeneration and loss of the extracellular matrix (ECM), is the main characteristic of OA pathogenesis. This review summarizes the biological mechanisms unraveled for circRNAs in recent years and discusses their involvement in OA pathogenesis, including their multiple forms of action inside and outside the cell.

## The formation, properties, and biological functions of circRNA

### Formation and properties of circRNA

**Figure 1:**
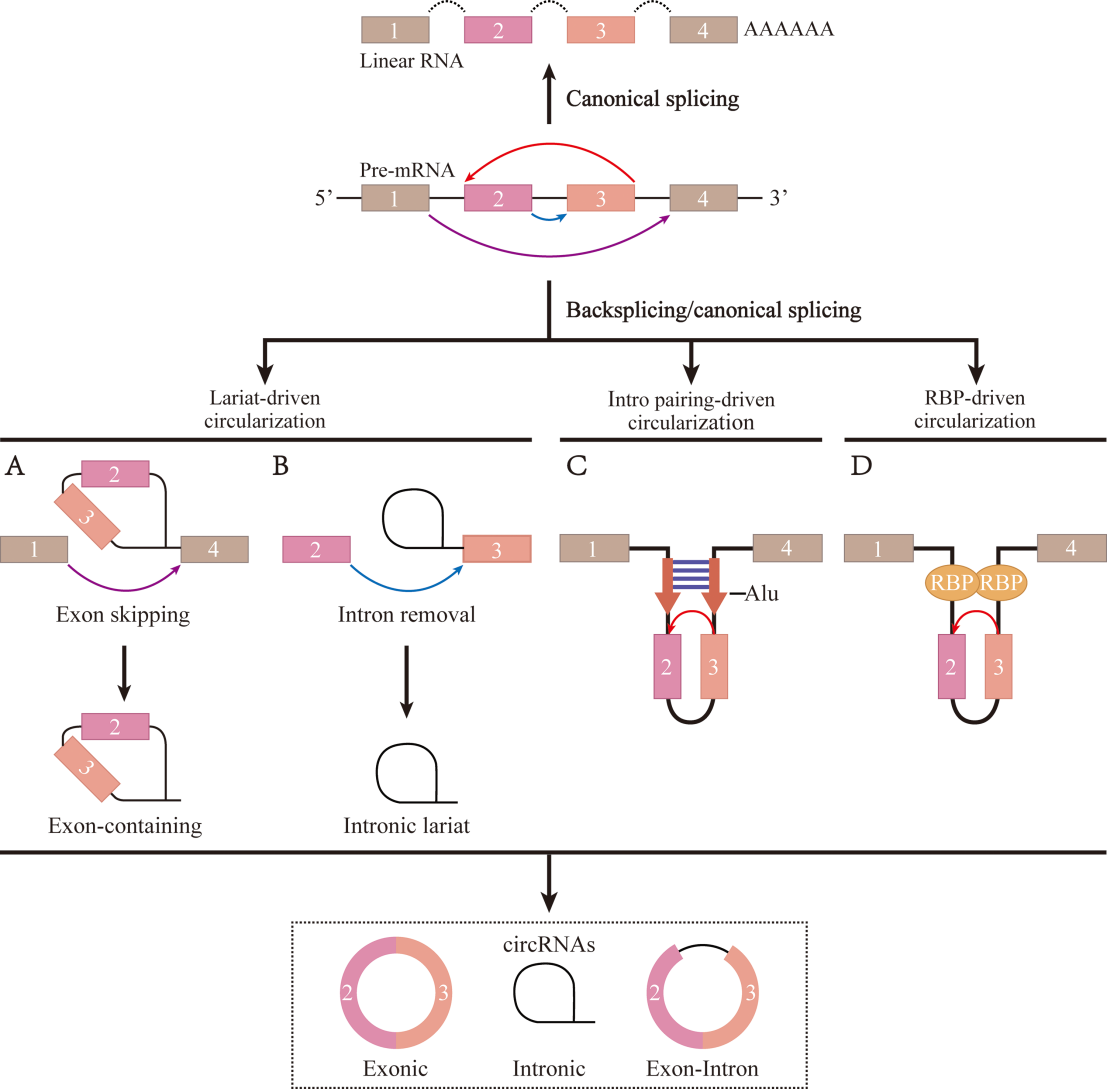
The formation process of circRNA. (**A**) The ribosomal protein assembles at the 5' and 3' splice site of the cyclized exon to form a lariat structure, causing exon skipping and driving circularization. (**B**) To form a mature circRNA, the intron relies on reverse splicing of the motif sequences at the 5' and 3' ends to drive circularization, followed by shearing. (**C**) The flanking introns of the cyclized exon forme parallel side-by-side duplexes through base-complementary pairing to drive circularization. (**D**) RNA-binding protein promotes exons circularization by binding to flanking intron sequences.

CircRNAs are a specific type of closed-loop endogenous non-coding RNA. CircRNAs do not contain either a 5′ cap or a 3′ poly A-tail and are produced by reverse splicing of a precursor mRNA (8). The generation of circRNAs can be summarized by the covalent linking of the 5′ splice site of a downstream intron to the 3′ splice acceptor site of an upstream intron (9). CircRNAs can be divided into three main categories based on their mode of formation (Figure 1): exonic circRNAs (EciRNAs), exon-intron circRNAs (EIciRNAs), and circular intronic circRNAs (ciRNAs) (10). Most circRNAs are EciRNAs, which are single-stranded non-collinear molecules composed of single or multiple exons (11). CircRNAs are expressed at much lower levels than linear RNAs. Consequently, The biological relevance of circRNAs was underestimated until advances in next-generation sequencing technology were introduced for their efficient detection. CircRNAs have the following properties: rich content, high stability, high conservation, and tissue specificity (12, 13). Based on these characteristics, it is reasonable to speculate that circRNAs might be ideal biomarkers for certain diseases, as well as novel therapeutic targets with clinical translational research potential.

### Biological functions of circRNA

#### MiRNA sponges

According to the cytoplasmic localization and cell stability of circRNAs, many studies have pursued the idea that circRNAs might function as competing for endogenous RNAs (ceRNAs). The antisense cerebellar degenerative-related protein-1 (CDR1as) gene was the first gene reported to produce circRNA molecules. CircCDR1as contains more than 70 selectively-conserved miRNA target sites that strongly inhibit miRNA activity, proving that circRNAs could function as miRNA sponges (14). CDR1as silences miR-7 by directly binding to it, resulting in the upregulation of miR-7 downstream target gene expression, whereas knockdown of circCDR1as results in the downregulation of miR-7 downstream target genes such as phosphatidylinositol 3-kinase (PI3K) (15). CircRNAs have shown potential as miRNA sponges, but their practical use remains problematic. Despite the widespread presence of circRNAs in nature, most mammals have low levels of circRNA, accounting for only 5%–10% of linear RNA, implying relatively fewer miRNA-binding sites (16) and limiting their use as biomarkers.

#### Transcriptional regulation

CircRNAs are mainly present in the cytoplasm as exons. However, other types of circRNAs with distinct properties deserve mention. Both ciRNAs and EIciRNAs tend to be enriched in the nucleus to promote RNA Pol II transcription. For example, the ciRNA, ci-ankrd52, binds RNA Pol II to promote gene transcription, and its knockdown results in reduced expression of its parental gene (17). EIciRNAs such as CircEIF3J and CircPAIP2, which are also present in the nucleus, bind to the U-small ribonucleoprotein and then to pol II to similarly regulate gene expression (18). Additionally, many studies have shown that ciRNAs and EIciRNAs may have cis-regulatory effects on the transcription of the coding gene (19).

#### Protein sponge/decoy

CircRNAs can be involved in RNA-binding protein (RBPs)-related activities, such as acting as super sponges to influence RBP synthesis. For example, circMBL acts as a protein sponge to ensure effective negative feedback loop. When mannose-binding lectin (MBL) protein expression levels are too high, MBL can act as an RBP binding to flanking introns and thus promote circMBL production. This mechanism prevents additional linear MBL mRNA and protein expression (20). However, circRNAs-protein interactions do not fully inhibit protein function, as they can only act as protein scaffolds to co-regulate gene expression. For example, CircAmotl1 can act as a protein scaffold for PI3K and AKT serine/threonine kinase 1 (AKT1), leading to AKT1 phosphorylation and promotion of a cardioprotective nuclear translocation of pAKT in neonates (21). Compared to the ceRNA mechanism, the two unique protein binding functions of circRNAs might be a new direction in the field of circRNA reseach.

#### Independent translation

In recent years, circRNAs have been found to be independently translatable by two mechanisms, despite the lack of a 5′ cap structure (22). One of their translation mechanisms involves the internal ribosomal entry site (IRES), which is a relatively short RNA-sequence segment that mediates ribosome binding to RNA without relying on the 5′ cap (23). For example, circFBXW7 contains an open reading frame (ORF) initiated by IRES that allows translation initiation, independent of the 5′-cap structure. This translation increases F-box and WD-repeat domain-containing 7 (FBXW7) expression and induces c-Myc ubiquitination degradation in triple-negative breast cancer (24). A second mechanism involves an N6-methyladenosine (m6A)-dependent form of circRNAs that can be translated even without IRES sequences. m6A methylation, at the 6th N position of the RNA adenylate, is a dynamic and reversible modification. Yang et al. identified 499 m6A-related circRNAs, of which 25 were validated by circRNA-m6A-seq (25). m6A-driven circRNA translation was observed to be enhanced by methyltransferase-like 3 (METTL3) and methyltransferase-like 4 (METTL14) complex and demethylated by fat mass and obesity-associated protein (FTO) (25). Many studies have suggested that the translational function of circRNAs may be common in the human transcriptome. However, based on the circular-specific structure of circRNAs, whether the novel pattern of circRNA translation is IRES- or m6A-mediated, still needs to be discussed with caution.

## Multiple mechanisms of circRNA-mediated regulation of OA

### CircRNA regulates multiple phenotypes of OA in the form of ceRNA

#### Inflammatory

**Figure 2:**
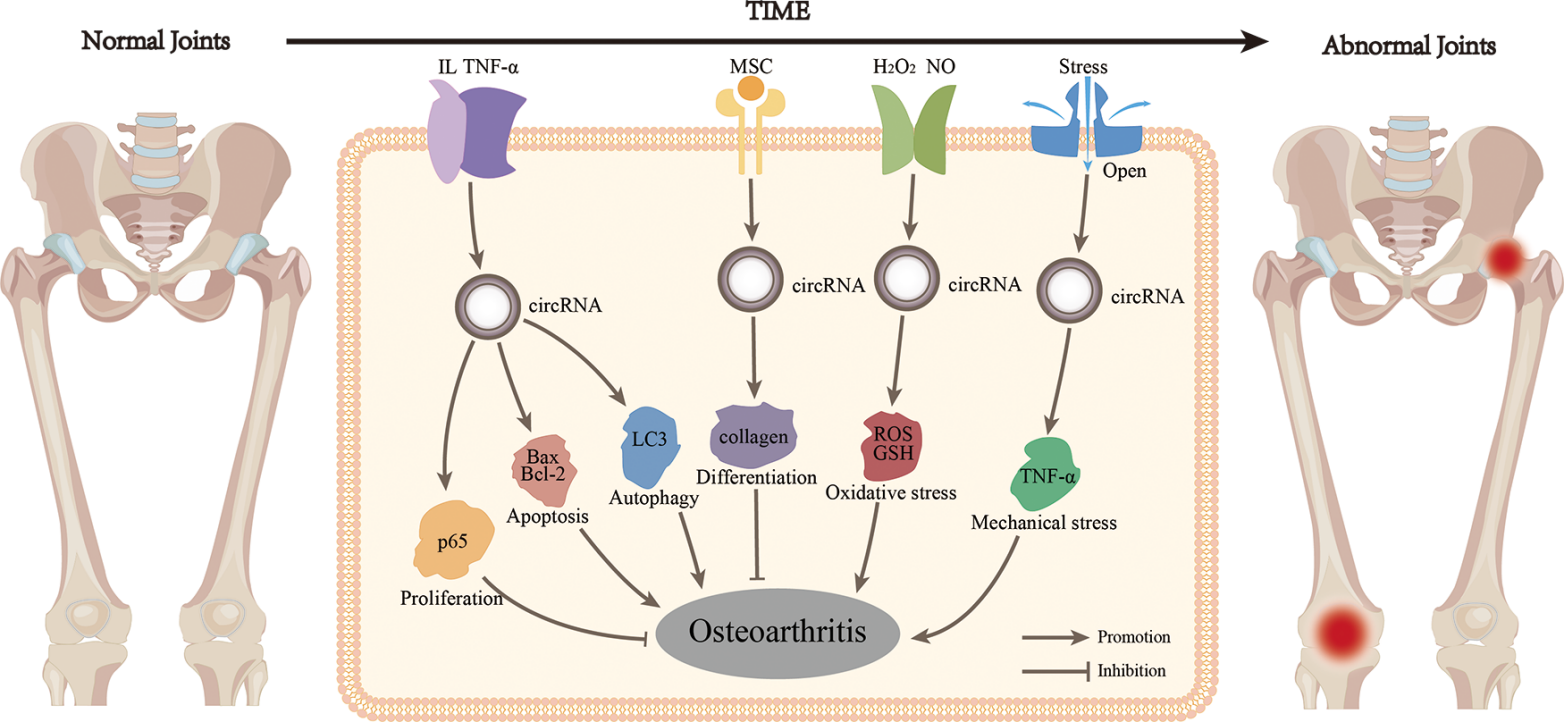
CircRNA regulates multiple phenotypes of OA in several ways. CircRNA activated by inflammatory factors could participate in proliferation, apoptosis and autophagy, thus regulating OA. CircRNA could also participate in the process of OA through differentiation, oxidative stress and mechanical stress alone.

Previously, OA was thought to be the result of only anatomical and functional joint injuries caused by cartilage degeneration. However, in recent years, the idea that inflammatory mediators produced by synovial, cartilage, and subchondral bone cause the eventual pathogenesis has been increasingly recognized (26). Multiple inflammatory cells are recruited to the synovial joints including T and B cells. The interaction between those immune cells and other joint cells might constitute a vicious cycle (27). During the early stages of OA, chondrocytes are activated in a compensatory manner to enhance ECM synthesis. However, they also produce and release pro-inflammatory cytokines, such as tumor necrosis factor-α (TNF-α) and interleukin (IL). Inflammatory factors damage cells and the ECM, whereas cell breakdown products stimulate inflammation. CircRNAs mediate inflammatory reactions through ceRNA-mediated mechanisms and participate in OA by combining proliferation and apoptosis, autophagy, oxidative stress, and mechanical stress (Figure 2). For example, circ_0134111 was overexpressed in the CHON-001 cartilage cell line and in an OA rat model induced by IL-1β. It is also a sponge for miR-224-5p to inhibit the C–C motif chemokine ligand 2 (CCL2) gene and promote the synthesis of IL-6 and TNF-α (28). Newly synthesized inflammatory factors continue to degrade the ECM and destroy cells (28). CircVMA21 expression was also decreased in IL-1β-stimulated C28/I2 chondrocytes, and the expression of IL-6, TNF-α, and prostaglandin E2 was enhanced through the miR-495-3p/FBWX7 axis, which led to apoptosis and cartilage degeneration (29). On IL-1β-stimulated chondrocytes, reduced circ0022383 expression enhanced IL-6 and TNF-α synthesis (30), whereas reduced IL-17-dependent circCDRas1 expression (15), with subsequent apoptosis in both cases. In addition, overexpression of IL-6 on IL-1β-stimulated chondrocytes activated the release of the pro-inflammatory cytokine IL-17, which exacerbated OA (31). Caso et al. found that IL-6 and IL-17 were both significantly activated in rheumatoid and psoriatic arthritis, respectively, suggesting that they may be common factors in inflammatory arthropathies (32). Another study showed that IL-1β, IL-6, and IL-17 expression in synovial fibroblasts of OA patients (33). Therefore, we speculate that most circRNAs involved in OA chondrocyte injury are triggered by the expression of pro-inflammatory factors such as IL-1β, IL-6, IL-17, and TNF-α, which eventually lead to the OA progression.

#### Proliferation and death

A typical feature of OA is that cells stop proliferating and die, which is accompanied by ECM degradation. ECM homeostasis could ensure that articular cartilage maintain a certain functionality, but OA patients are unable to provide such functionality due to a reduction in aggrecan and collagen in the ECM, which in turn causes the collapse of the joint surface. Although inflammatory reactions are the main cause of OA, many circRNAs could participate in OA onset alone without the need for pro-inflammatory factors as circRNAs regulate both proliferation and cell death. For example, lymphoid enhancer factor-1 (LEF-1) acts as a forward transcription factor in primary chondrocytes of OA patients, degrades the ECM *via* the LEF1/CircRNF12/miR-655/myeloid differentiation factor 88 (MyD88) pathway and reduces the synthesis of the p65 protein. After establishing a model of destabilization of the medial meniscus (DMM) in Sprague-Dawley (SD) rats, CircRNF121 was found to inhibit proliferation and promote apoptosis in the absence of inflammation, thereby exacerbating OA progression (34). CircRNAs can also induce apoptosis as illustrated by the fact that circCTNNA1 expression decreased in OA, incapacitating its association with miR-29a, leading to a decrease in the BCL2 apoptosis regulator (Bcl-2) and an increase in the apoptotic BCL2-associated X apoptosis regulator (BAX, this resulting in synovial cell apoptosis and directly induced diseases (35). Interestingly, circRNAs do not only act on chondrocytes in OA. For example, a reduced expression of circ_0005567 failed to prevent polarization of M1 macrophages by ceRNA-dependent mechanisms. This is important as activated macrophages secrete large amounts of pro-inflammatory factors that indirectly promote chondrocyte apoptosis and accelerate the pathological process of osteoarthritis (36). In previous studies, circRNA changed chondrocyte viability mainly through apoptosis; however, it could also be through autophagy. For example, circRHOT1 enhanced the expression of the G1/S-specific cell cycle protein-D1 (CCND1) by sponging miR-142-5p to decrease the expression of the autophagy-related gene microtubule-associated protein 1 light chain3 (LC3), and then inhibits cellular autophagy to promote proliferation(37). Similar results were obtained from an anterior cruciate ligament transection (ACLT) model in SD rats (37). Although apoptosis and autophagy are mutually exclusive processes, both apoptosis and autophagy cause cell death. However, moderate autophagy can promote cell survival (38). Autophagy is essential for the degradation of proteins that tend to aggregate and dysfunctional organelles, such as mitochondria (39). So, in cases where both autophagy and apoptosis may occur, autophagy should be given more importance For example, circFOXO3 overexpression alleviates OA by activating the forkhead box O3 (FOXO3) parental gene and thus the autophagy pathway, which in turn impairs chondrocyte apoptosis and promote ECM anabolism (40). Conversely, CircMELK exacerbated OA by regulating the miR-497-5p/MYD88/nuclear factor-kappa B (NF-κB) signaling axis, promoting chondrocyte apoptosis and inhibiting autophagy (41). Although circMELK and circFOXO3 are expressed at different levels in OA, their effects promote apoptosis and inhibit autophagy through ceRNA mechanisms, ultimately leading to OA progression. This may be related to the possibility that IL-1β initially stimulates autophagy in chondrocytes, while significantly inhibiting autophagy and activating apoptosis in late OA (31). These examples suggest that the major means to alleviate OA are cell proliferation and ECM synthesis, while at the same time enhancing chondrocyte autophagy to inhibit apoptosis. However, the idea of a therapeutic intervention based on circRNAs needs to be tested in future studies.

#### Cell differentiation

With the development of regenerative medicine technology, stem cells have been widely used in the treatment of various diseases, including cartilage degenerative diseases, mainly through the direct repair of damaged tissues through multidirectional differentiation potential. Stem cells are transformed into osteoblasts and chondrocytes under specific conditions *in vivo* and *in vitro* to repair the articular cartilage (42). CircRNAs are heavily involved in cell differentiation in the musculoskeletal system, especially in pluripotent stem cell differentiation. For example, CircMYL1 inhibits proliferation and promotes differentiation of myoblasts (43), and CDR1as promotes adipogenesis and inhibits osteogenic differentiation of bone marrow mesenchymal stem cells (BMSCs) in the femoral head osteonecrosis (44). The involvement of circRNAs in the chondrogenic differentiation of BMSC has also been observed in OA. CircZC3H7B overexpression promotes the induction of cartilage-specific genes and proteins through the miR-3677-3p/Sox9 axis, allowing BMSC to differentiate into chondrocytes, which might reduce ECM degradation and enhance collagen synthesis, thereby inhibiting OA progression (45). Other types, such as circRNAs in adipose stem cells (ADSC) have been described to regulate OA. For example, circATRNL1 and circNFIX overexpression enhance the chondrogenic differentiation ability of ADSC (46). CircNFIX acts as a miR-758-3p sponge, targeting the miR-758-3p/lysine demethylase 6A (KDM6A) axis to prevent chondrocyte degradation and attenuate OA in DMM mice, thereby playing a role in cartilage differentiation and chondrocyte degeneration (47). Based on the multidirectional differentiation potential of stem cells, the involvement of CircRNA in stem cell differentiation and their secreted exosome regulatory mechanisms have become widely studied topics in cartilage regeneration research. Under certain conditions, circRNAs could induce stem cells to differentiate into chondrocytes, thereby maintaining their function and stability.

#### Oxidative stress

Tissue damage can occur when oxidation levels exceed the cellular clearance capacity. The ECM is a hypoxic environment owing to the lack of nerves and blood vessels. As chondrocytes in the ECM are the only cells that form articular cartilage, the loss of chondrocytes under oxidative stress is the main cause of OA (48). For example, increased levels of circ_0136474 in CHON-001 cells decrease the synthesis of reduced glutathione (GSH) and superoxide dismutase through the miR-766-3p/DNA methyltransferase 3 alpha (DNMT3A) pathway, which corresponds to increased reactive oxygen species (ROS) clusters and malondialdehyde as a result of lipid peroxidation. Thus, cells subjected to sustained oxidative stress, undergo accelerated metabolism, cessation of cell proliferation, and apoptosis (49). When mimicking oxidative stress *in vitro* in primary chondrocytes by the addition of peroxidase, CircRSU1 competitively binds to miR-93-5p and promotes the expression of mitogen-activated protein kinase kinase kinase 8 (MAP3K8). In a DMM model in C57BL/6 mice, the knockdown of CircRSU1 resulted in a significant reduction in cyclooxygenase-2 and inducible nitric oxide synthase levels and the inhibition of oxidative stress while causing enhanced inflammation and metabolism (50). The construction of OA-damaged C28/I2 cell lines resulted in reduced circLRP1B expression and induction of multiple oxidative markers and apoptosis (51). These studies showed that circRNAs are involved in a series of oxidative stress regulatory processes in OA. For example, enhanced catabolism of the ECM induced by oxidative stress is accompanied by an abnormal inflammatory response that dramatically alters the proliferation and death phenotype of cells, suggesting that oxidative stress is a key factor in the development of OA. Several antioxidant drugs have been shown to be effective in treating OA. Quercetin, a novel antioxidant protective drug for chondrocytes, significantly ameliorates oxidative stress-induced apoptosis and ECM degradation(52). Glabridin, a strong antioxidant, protects chondrocytes from oxidative stress-induced apoptosis and increases autophagy to protect articular cartilage from damage in rats with OA (53). These evidences support the idea of a preference for autophagy vs. apoptosis, as discussed above. Unfortunately, no studies have identified circRNAs associated with antioxidant drugs for the treatment of OA.

#### Mechanical stress

**Figure 3:**
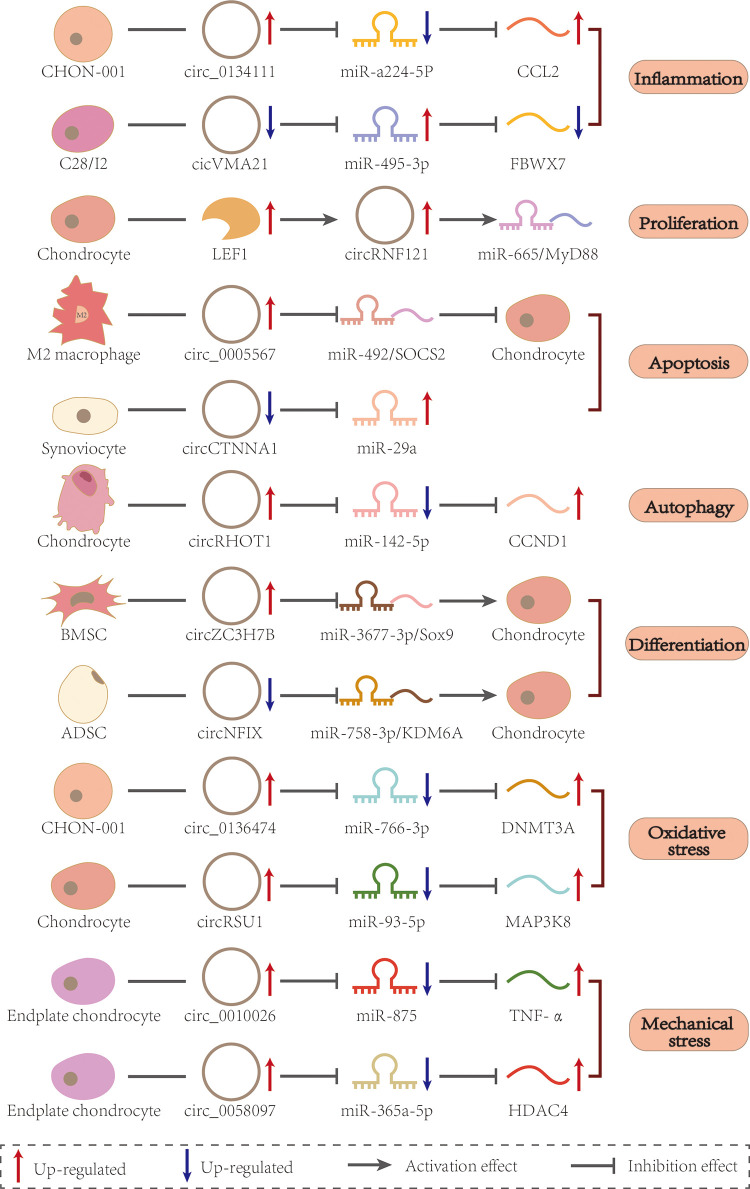
CircRNAs in different cells regulate miRNAs and mRNAs *via* the ceRNA mechanism. Each of the four types of arrows has a different meaning, as shown in the label at the bottom of the figure.

Articular cartilage, that consists mainly of chondrocytes surrounded by synovial fluid and ECM, allows for motion cushioning and pressure absorption (54). An accumulation of joint load, such as weight gain caused by obesity and changes in body composition, significantly increases the risk of OA (55). Pressure-sensitive ion channels are present on the surface of chondrocytes, so an increased mechanical load may open the channels, making the articular cells more sensitive to mechanical stress, mostly when inflammatory signals are present. For example, in OA chondrocytes, the transient receptor potential channel vanilloid 4 (TRPV4), increased chondrocyte apoptosis by Ca2+-mediated mechanical stress (56). It has also been observed that excessive mechanical tension eventually leads to genetic changes in endplate chondrocytes and consequent cartilage degeneration (57). Indeed, tension-sensitive circ_0010026 was detected in human endplate chondrocyte with increased mechanical stress, leading to ECM degradation and degeneration of endplate chondrocytes *via* the miR-875/TNF-α axis (58). Circ_0058097 is derived from fibronectin1 (FN1). The protein product of the FN1 gene mediates chondrocyte migration and adhesion. When mechanical stress is applied to endplate chondrocytes, circ_0058097 expression increases, inhibiting proliferation and inducing apoptosis *via* the miR-365a-5p/histone deacetylase 4 (HDAC4) axis (59). Previous studies on mechanical stress have paid more attention to changes in mRNAs and protein levels. These examples showed the same effective increase in stress resistance of endplate chondrocytes after altering circRNA expression levels, as well as a reduction in apoptosis. It is suggested that circRNA involvement in mechanical stress regulation might be an important trigger for the degeneration of endplate chondrocytes. However, mechanical stress may not be an absolute disadvantage. Yang et al. found that moderate mechanical stimulation could alleviate cartilage degeneration and chondrocyte damage by inhibiting the tumor necrosis factor-related apoptosis-induced ligand (TRAIL)/NF-κB/nucleotide-binding and oligomerization domain-like receptor containing protein 3 (NLRP3) signaling pathway (60). It is possible that moderate mechanical stress reduces IL-1β-induced chondrocyte apoptosis by maintaining mitochondrial function by clearing ROS and activating autophagy, whereas excessive mechanical stress might result in strong mitochondrial dysfunction and apoptosis (61). Therefore, circRNAs combined with moderate stimulation may provide a new avenue for the prevention and treatment of mechanical stress-induced endplate cartilage degeneration (Figure 3 and Table 1).

**Table 1:** CircRNAs involved in OA progression by intracellular ceRNA mechanism.

CircRNA	Host gene	Expression	Function	Sponge	Target	Model	Years	Reference
circ_0134111	PDE1C	↑	Inflammation	miR-224-5p	CCL2	DMM (SD rat)	2021	(28)
cicVMA21	VMA21	↓	Inflammation	miR-495-3p	FBWX7	DMM (SD rat)	2022	(29)
circRNF121	RNF121	↑	Proliferation	miR-665	MyD88	DMM (SD rat)	2020	(35)
circCTNNA1	CTNNA1	↓	Apoptosis	miR-29a	—	—	2022	(36)
circ_0005567	EPS15	↑	Apoptosis	miR-492	SOCS2	—	2021	(37)
circRHOT1	RHOT1	↑	Autophagy	miR-142-5p	CCND1	ACLT (SD rat)	2022	(38)
circZC3H7B	ZC3H7B	↑	Differentiation	miR-3677-3p	Sox9	DMM (C57BL/6 mouse)	2021	(46)
circNFIX	NFIX	↓	Differentiation	miR-758-3p	KDM6A	DMM (C57BL/6 mouse)	2022	(47)
circ_0136474	ASH2L	↑	Oxidative stress	miR-766-3p	DNMT3A	—	2021	(50)
circRSU1	RSU1	↑	Oxidative stress	miR-93-5p	MAP3K8	DMM (C57BL/6 mouse)	2021	(51)
circ_0010026	PDPN	↑	Mechanical stress	miR-875	TNF-α	—	2017	(59)
circ_0058097	FN1	↑	Mechanical stress	miR-365a-5p	HDAC4	—	2020	(60)

### CircRNA participates in the regulation of OA in other forms

#### CircRNA binding to RBP

**Figure 4:**
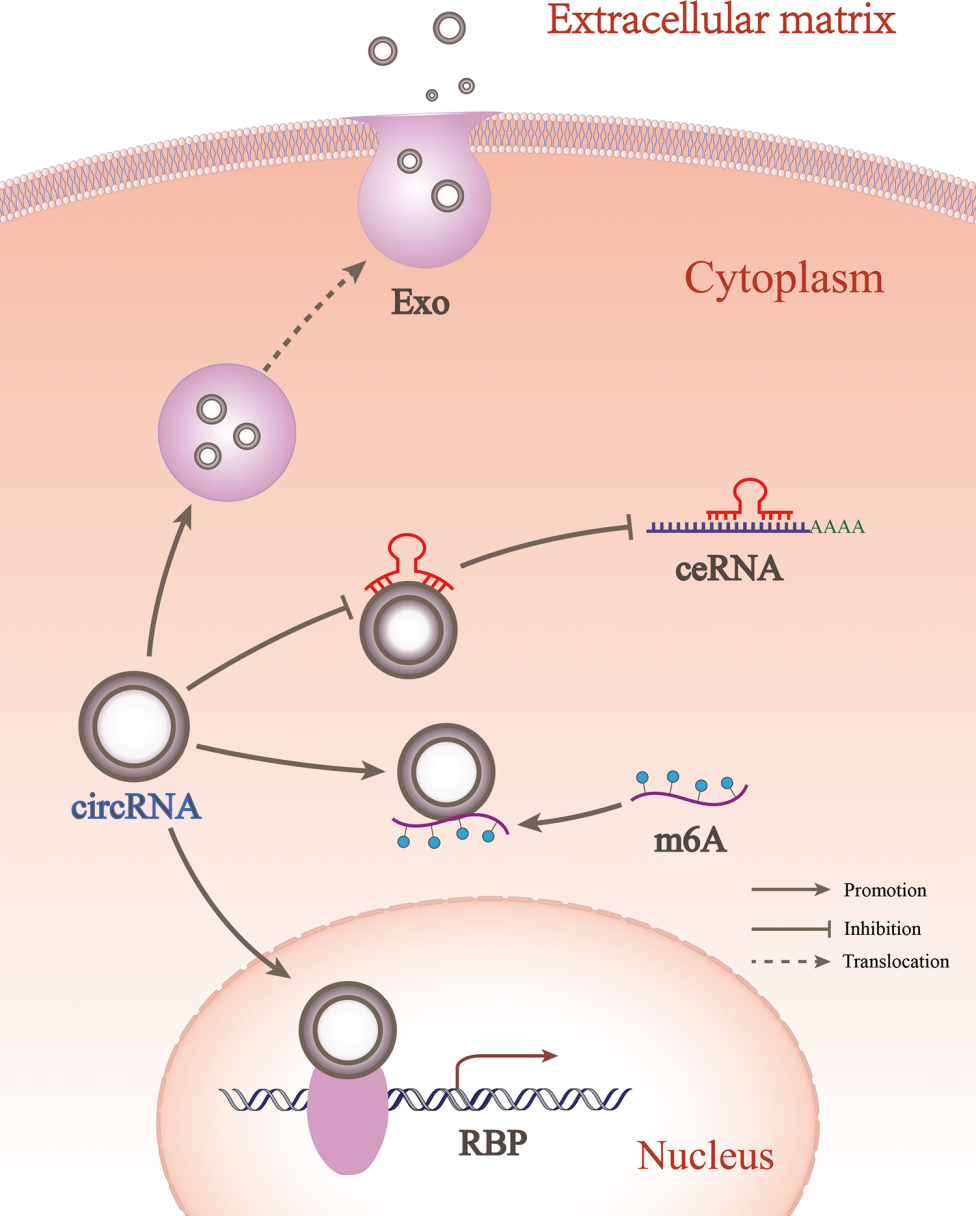
CircRNA participates in OA in different forms. circRNA could regulate OA between cells through exosomes, while within cells it could regulate OA through ceRNA, RBP, and m6A forms.

CeRNA is the most important type of circRNA. However, there are small numbers of circRNAs that impact OA progression *via* other newly discovered mechanisms, such as RBP, m6A, and exosomes (Figure 4). RBP controls the RNA processes of shear editing, translational regulation, and translational targeting by interacting with RNA, especially non-coding RNA (62). CircRNAs bind proteins or act as linking scaffolds for multiple proteins, mainly affecting transcription, translation, and protein degradation (63). RBP binding in the circRNA mode may be extensively involved in disease progression in the musculoskeletal system. For example, when CircLRP6 is highly expressed, it acts as a protein scaffold to bind to LSD1 and EZH2. This facilitates the binding of transcription factors to promoter regions, thereby inducing proliferation and metastasis of osteosarcoma cells (64). Circ_0066523 inhibits phosphatase and tensin homolog (PTEN) genes *via* epigenetic inheritance, thereby activating the PI3K/AKT pathway to promote BMSC proliferation and differentiation and effectively alleviating osteonecrosis of the femoral head (65). CircStag1 binds to the HuR protein and promotes its translocation into the cytoplasm, leading to the activation of the Wnt signaling pathway to promote bone tissue regeneration. This mechanism provides a strategy to prevent bone metabolism disorders, such as postmenopausal osteoporosis (66). In OA, CircPDE4B acts as a linking scaffold to midline 1 (MID1) and RIC8 guanine-nucleotide exchange factor A (RIC8A), allowing their direct binding to form a ternary complex that allows RIC8A to be degraded *via* the ubiquitin-proteasome pathway, thereby preventing cartilage degeneration (67). In recent years, research on OA and circRNA relevance has focused on miRNA sponge mechanisms, with less research on RBP mechanisms. In fact, circRNAs may be involved in both the ceRNA- and RBP-mediated mechanisms. For example, circTHBS1 engages miR-204-5p in a ceRNA-dependent way to promote the expression of inhibin subunit Beta A (INHBA). CircTHBS1 can also bind HuR in an RBP-dependent manner to enhance INHBA mRNA stability and ultimately promote gastric cancer progression (68). However, such combined mechanisms have not been identified in OA and require further investigation. The finding of CircPDE4B suggests that circRNAs research in OA will not be limited to the sponge mechanism of miRNAs, and should be extended to an in-depth study of their direct interactions with RNA-binding proteins.

#### CircRNA involved in m6A

m6A is the most common modification in mRNA and ncRNAs, accounting for over 60% of all RNA modifications. m6A modification refers to the dynamic and reversible methylation at the 6th N position of the RNA adenylates. The regulatory factors of the m6A mechanism include methylation transferases, demethylases, and methylated reading proteins, defined as writers, erasers, and readers, respectively. m6A modifications have been found to occur on miRNAs, lncRNAs, and circRNAs, which are widely involved in a variety of bone and joint diseases (69). For writers, such as Methyltransferase METTL3, the main role is to catalyze the m6A modification of adenosine on RNA. METTL3 increases the expression level of CircNRIP1 through m6A modification, while induction of CircNRIP1 exerts oncogenic effects in osteosarcoma by sponging miR-199a (70). Erasers, such as demethylase alkB homolog 5 (ALKBH5), act to demethylate bases that have been modified by m6A. ALKBH5 reversed osteoclast differentiation and subsequent bone resorption induced by circ_0008542 (71). m6A modifications of circRNAs also exist in OA. For readers, such as the methylation reading protein YTH N6-methyladenosine RNA-binding protein 2 (YTHDF2), the main function is to recognize bases on RNA sequences that undergo m6A modification, thereby activating RNA degradation. YTHDF2 recognizes the m6A site of circRERE and leads to its degradation (72). This alters the expression level of circRERE, involved in the ceRNA mechanism, thus allowing the downstream circRERE/miR-195-5p/interferon regulatory factor 2 binding protein-like (IRF2BPL) axis to be repressed. This repression leads to chondrocyte apoptosis, significantly promoting OA progression (72). In summary, m6A regulation of circRNA occurs through the alteration of its own activity, unlike ceRNA and RBP modes of action. While current studies of m6A in OA-related circRNAs are focused on reader mode, further studies of writers and erasers are worth exploring. Moreover, circRNAs not only participate in m6A, but also alter the methylation of downstream target genes to mediate OA. Zhang et al. found that overexpression of circFADS2 significantly increased methylation of the miR-195-5p parental gene and inhibited gene expression, which in turn impaired chondrocyte apoptosis (73). However, this evidence could not sufficiently prove the ability of circRNAs to methylate downstream genes, so further studies are needed. Previously, the participation of circRNAs in m6A models was less significant. Therefore, understanding the role of m6A-modified circRNAs in the pathological progression of OA could provide insight into the overall physiological mechanisms of the disease and promote the development of new biomarkers.

#### CircRNA involved in exosomes

Exosomes have a bilayer membrane structure that detaches from the cell membrane or is secreted by the cell. They are nanoscale membrane vesicles actively released by cells. They possess strong intercellular transport capacity that mediates the intercellular information exchange (74). Stem cell-derived exosomes tend to contain higher levels of growth factors and have a good effect on tissue repair. Many advances have been made in the direction of identifying miRNAs and lncRNAs in stem cell exosomes for the treatment of OA. For example, umbilical cord mesenchymal stem cell (UMSC) exosomes contain miR-148a and miR-29b, which promote cartilage regeneration and suppress inflammatory responses in rats (75). UMSC exosomes also contain lncRNAH19, which inhibits apoptosis by promoting chondrocyte migration and matrix synthesis through the miR-29b-3p/FOXO3 axis (76). The involvement of intercellular exosome-containing circRNAs in OA has recently been explored. After the addition of BMSC-derived circHIPK3-containing exosomes, C57BL/6 mouse chondrocytes promoted ECM synthesis and inhibited apoptosis *via* the circHIPK3/miR-124-3p/myosin heavy chain 9 (MYH9) axis (77). DMM mice injected with exosomes containing circZC3H7B reduced the extent of OA *via* the miR-3677-3p/Sox9 axis (45). Finally, SD rats injected with exosomes containing circRNA3503, isolated from synovial mesenchymal stem cells from the joint cavity, showed improved proliferation and better controlled OA progression (78). Exosomal MSC-derived circPARD3B was also found in the serum of patients with OA, which increased the expression of vascular endothelial growth factor (VEGF) through miR-326 consequently inducing synovial angiogenesis (79). This effect might be one explanation for the inflammatory synovial hyperplasia often associated with patients with OA (79). These exosomes improve chondrocyte viability and various phenotypes mainly through the intercellular ceRNA mechanism, thereby alleviating OA. A better understanding of the intercellular role of circRNAs in OA may show potential for the development of new treatments for OA. Furthermore, chondrocytes that have entered the injury stage can also produce exosomes that cause pathological processes in other adjacent normal or pathologic chondrocytes. lncRNA OANCT exosomes from damaged chondrocytes, which activates the PI3K/AKT mechanistic target of rapamycin kinase (mTOR) pathway, exacerbates OA performance, promotes M1 polarization after entry into M0, and enhances the production of inflammatory factors in the rat cells (80). Similar findings have been reported on circRNA. Inflammatory factors induce normal CHON-001 chondrocytes to produce exosome Circ_BRWD1, which causes an inflammatory response and abnormal proliferation and apoptosis in other chondrocytes *via* the miR-1277/TNF receptor-associated factor 6 (TRAF6) axis (81). Pathological CHON-001 cells also produce exosomes containing Circ_0001846, which causes inflammation and ECM degradation to accelerate OA progression *via* the miR-149-5p/Wnt family member 5 B (WNT5B) axis (82). Further examples illustrate the role of exosomes depending on the nature of the donor cells. MSC-derived circRNAs exosomes are therapeutically useful for restoring the structural and functional integrity of joints. However, damaged chondrocytes can also produce circRNAs exosomes to stimulate the deficient development of surrounding OA cells. Although exosomes have advantages in tissue regeneration, they still have several disadvantages, such as low yield, weak function, and low specificity, possess disadvantages that cannot meet the quantitative and qualitative needs in clinical settings. In this sense, further research and translational efforts are required for specific clinical applications.

## Conclusions

OA is a widespread joint disease that severely affects patients' quality of life, with no specific drugs or diagnostic methods for its early treatment as the mechanisms of OA progression, remain unclear. CircRNAs, are novel markers in OA and have a potential clinical value in OA development. This review discusses the occurrence, characteristics, and functions of circRNAs, focusing on multiple forms of regulation of circRNA in OA. The inflammatory response is the most dominant manifestation of OA. CircRNAs are involved in OA *via* inflammation, in combination with proliferation, apoptosis, autophagy, differentiation, oxidative stress, and mechanical stress. Most circRNAs contribute to OA pathogenesis, mainly in the form of intracellular miRNA sponges in chondrocytes, endplate chondrocytes, mesenchymal stem cells, synoviocytes, and macrophages. However, a small number of circRNAs are also involved in OA in other ways, such as RBP, m6A, or intercellular exosomal mechanisms, including ECM metabolic imbalance, local tissue inflammation, and multidirectional cell differentiation. CircRNAs are stable, have a rich source of specimens, are readily available in cartilage and synovial fluid, and can be isolated from peripheral blood in form of microvesicles and exosomes. Their great potential suggests that they might be a new tool for the overall palliation rather than a purely point-to-point treatment of OA. However, there is an urgent need for a common standardized nomenclature to facilitate research. With the gradual discovery of new mechanisms, circRNAs might enable the development of different strategies for the early diagnosis and etiological treatment of OA in the future.

## Data Availability

The data supporting this review are from previously reported studies and datasets, which have been cited.
